# Curcumin analog HO‐3867 triggers apoptotic pathways through activating JNK1/2 signalling in human oral squamous cell carcinoma cells

**DOI:** 10.1111/jcmm.17248

**Published:** 2022-02-21

**Authors:** Chi‐Wei Chen, Ming‐Ju Hsieh, Po‐Chung Ju, Yi‐Hsien Hsieh, Chun‐Wen Su, Yen‐Lin Chen, Shun‐Fa Yang, Chiao‐Wen Lin

**Affiliations:** ^1^ Department of Life Science College of Science and Engineering National Dong Hwa University Hualien Taiwan; ^2^ Oral Cancer Research Center Changhua Christian Hospital Changhua Taiwan; ^3^ Department of Post‐Baccalaureate Medicine College of Medicine National Chung Hsing University Taichung Taiwan; ^4^ Graduate Institute of Biomedical Sciences China Medical University Taichung Taiwan; ^5^ School of Medicine Chung Shan Medical University Taichung Taiwan; ^6^ Department of Psychiatry Chung Shan Medical University Hospital Taichung Taiwan; ^7^ Institute of Medicine Chung Shan Medical University Taichung Taiwan; ^8^ Department of Medical Research Chung Shan Medical University Hospital Taichung Taiwan; ^9^ Institute of Oral Sciences Chung Shan Medical University Taichung Taiwan; ^10^ Department of Dentistry Chung Shan Medical University Hospital Taichung 402 Taiwan

**Keywords:** apoptosis, HO‐3867, JNK1/2, oral squamous cell carcinoma

## Abstract

Human oral squamous cell carcinoma (OSCC) is the common head and neck malignancy in the world. While surgery, radiotherapy and chemotherapy are emerging as the standard treatment for OSCC patients, the outcome is limited to the recurrence and side effects. Therefore, patients with OSCC require alternative strategies for treatment. In this study, we aimed to explore the therapeutic effect and the mode of action of the novel curcumin analog, HO‐3867, against human OSCC cells. We analysed the cytotoxicity of HO‐3867 using MTT assay. In vitro mechanic studies were performed to determine whether MAPK pathway is involved in HO‐3867 induced cell apoptosis. As the results, we found HO‐3867 suppressed OSCC cells growth effectively. The flow cytometry data indicate that HO‐3867 induce the sub‐G1 phase. Moreover, we found that HO‐3867 induced cell apoptosis by triggering formation of activated caspase 3, caspase 8, caspase 9 and PARP. After dissecting MAPK pathway, we found HO‐3867 induced cell apoptosis via the c‐Jun N‐terminal kinase (JNK)1/2 pathway. Our results suggest that HO‐3867 is an effective anticancer agent as its induction of cell apoptosis through JNK1/2 pathway in human oral cancer cells.

## 1 INTRODUCTION

Oral squamous cell carcinoma (OSCC) is the most common oral cavity cancer with over 90% of cases.^1^ Betel nut chewing, smoking and drinking are the most common risk factors for oral cancer in Taiwan.^2^, ^3^, ^4^ OSCC often develops regional and distant lymph node.^5^, ^6^ Although surgery, radiotherapy and chemotherapy have applied for the treatments of OSCC, the prognosis of OSCC is still poor due to the recurrence and resistance to treatments.^7^, ^8^ According to its high incidence and mortality, development of new and effective treatments for OSCC is an urgent and unmet goal.

Programmed cell death (apoptosis) is a process of eliminating cells to maintain cell population and the normal growth of an organism during development, ageing and DNA damage.^9^, ^10^ On the contrary, defects in apoptosis result in neoplastic cells survival as dysregulated cell proliferation, increased cell motility and tumour progression.^11^ Apoptosis is triggered through intrinsic, extrinsic and endoplasmic reticulum (ER) pathways.^12^, ^13^ Caspases drive apoptosis through activation of caspases initiator 8, 9 and 10 (initiators), caspases 3 and 7 (executioners), and caspases 1, 4 and 5 (inflammatorys).^10^, ^14^ The inhibitors of apoptosis proteins are known as the inhibitor of apoptosis protein (IAP) family, including cellular inhibitors of apoptosis 1, 2 (cIAP‐1 and cIAP‐2), X‐linked inhibitor of apoptosis (XIAP) and survivin prohibit death receptor‐mediated apoptosis through binding caspases.^15^, ^16^, ^17^, ^18^, ^19^ The mitogen‐activated protein kinase (MAPKs) (ERKs, JNKs and p38 signalling) also mediate progression of apoptosis; however, the role of MAPKs is relied on status of activated MAPKs, cell types, stimuli or cell stress.^20^ Among them, activation of apoptotic via JNKs is through transcriptionally upregulating pro‐apoptotic genes or phosphorylating mitochondrial pro‐ and anti‐apoptotic proteins.^21^

Curcumin analog HO‐3867 is an antioxidant and antiproliferative compound as it is also known as an antitumour agent through blocking the Janus kinase/signal transducer and activator of transcription (JAK/STAT3) pathway and downregulation focal adhesion kinase and fatty acid synthase in human breast cancer, ovarian cancer and human pancreatic cancer cells alone or in combined with cisplatin or doxorubicin.^22^, ^23^, ^24^, ^25^, ^26^, ^27^, ^28^, ^29^, ^30^, ^31^ In addition, HO‐3867 is also found to activate phosphatase and tensin homolog (PTEN) in human smooth muscle cells and in lung and heart tissues.^32^, ^33^ A more recent study found that HO‐3867 transcriptionally converts mutant p53 protein to active wild‐type p53 in cancer cells.^34^ Nevertheless, HO‐3867 is revealed to rescue suppression of placenta‐specific protein 1 (PLAC1) level in ovarian cancer cells.^35^

As OSCC has over 40% mutant rate of TP53^8^, ^36^ and mutant TP53 leads cancer progression,^37^ we aimed to explore whether HO‐3867 is capable of suppressing OSCC cell growth. We analysed its therapeutic effect on OSCC and to discover the inside mechanisms involved in HO‐3867 induced apoptosis and attempted to define its underlying mechanisms.

## 2 MATERIALS AND METHODS

### 2.1 Cell culture and HO‐3867 treatment

Being purchased from the American Type Culture Collection (Manassas, VA, USA) and the Japanese Collection of Research Bioresources (Osaka, Japan), the human OSCC SCC‐9 and HSC‐3 cells were supplemented with 10% FBS, 5 mL glutamine and 1% penicillin/streptomycin, and cultured in DMEM. HO‐3867 was dissolved initially in 100% DMSO to achieve a 100 mM stock solution of HO‐3867, and appropriate amounts of stock solution were subsequently added into the culture medium to achieve the indicated concentrations.

### 2.2 Microculture tetrazolium (MTT) assay

To obtain information regarding the effect of apoptosis induced by HO‐3867, we subjected 6.5 × 10^4^/mL SCC‐9 and HSC‐3 cells in 24‐well plates for 16 h and treated them with different concentrations (0, 2.5, 5, 10 and 20 µM) of HO‐3867 to assay the cell viability via MTT assay as described previously.^38^, ^39^

### 2.3 Flow cytometric analysis

To estimate the proportion of SCC‐9 and HSC‐3 cells in different phases of the cell cycle affected by HO‐3867, cellular DNA contents were measured via flow cytometry as stated previously.^40^ Briefly, we cultured 7.0 × 10^5^ SCC‐9 and HSC‐3 cells in 6‐cm dishes and treated them with different concentrations (0, 2.5, 5, 10 and 20 µM) of HO‐3867 for 24 h. After staining with PI, 7.0 × 10^5^ SCC‐9 and HSC‐3 cells in one Eppendorf tube, the cell cycle was analysed on a BD AccuriTM C6 Plus personal flow cytometer (BD Biosciences, San Jose, CA, USA).

### 2.4 Annexin V‐FITC apoptosis staining assay

We cultured 7.0 × 10^5^ SCC‐9 and HSC‐3 cells in one 6‐cm dish and treated them with different concentrations (0, 2.5, 5, 10 and 20 µM) of HO‐3867 for 24 h. Subsequently, SCC‐9 and HSC‐3 cells were harvested with trypsinization together with floating non‐viable cells. The FITC Annexin V Apoptosis Detection Kit I was performed as reported previously.^41^

### 2.5 Human apoptosis array (ARY009, R&D systems)

Human apoptosis array (ARY009, R&D systems) Kit was used to evaluate protein lysates of 1.5 × 10^6^ SCC‐9 cells/dish from vehicle‐ or 20 µM HO‐3867‐treated for 24 h according to the manufacturer's protocols (R&D Systems, Minneapolis, MN, USA).

### 2.6 Protein extraction and western blot analysis

To investigate the molecular mechanism further, the initiator and effector caspases and signalling pathways were detected using Western blot analysis. As described previously, 7.0 x 10^5^/dish SCC‐9 and HSC‐3 cells were cultured in 6 cm plates for 16 h and treated with different concentrations (0, 2.5, 5, 10 and 20 µM) of HO‐3867 for 24 h, and the total cell lysates of SCC‐9 and HSC‐3 cells were prepared.^42^, ^43^ Blots were then incubated with a horseradish peroxidase goat anti‐rabbit or anti‐mouse.

### 2.7 Statistical analysis

The SigmaStat 2.0 software package (Jandel Scientific, San Rafael, CA, USA) was applied for statistical analyses. Differences between untreated and HO‐3867‐treated groups were calculated by Student's *t*‐test, and a p value of <0.05 was considered statistically significant. Each experiment was done in triplicate at least (*n* ≥ 3) were performed.

## 3 RESULTS

### 3.1 Cytotoxicity of HO‐3867 in human oral squamous cell carcinoma SCC‐9 and HSC‐3 cells

**FIGURE 1:**
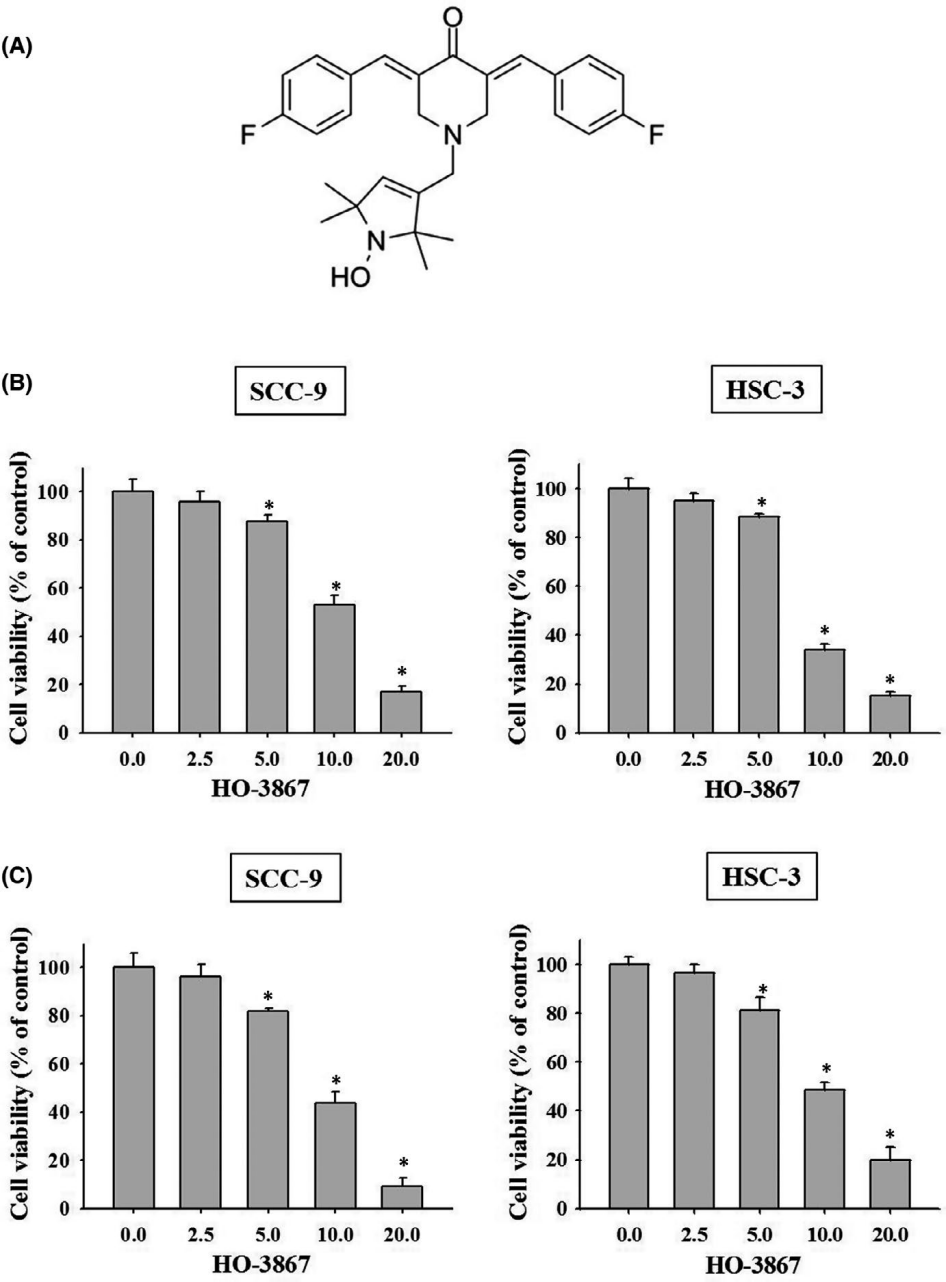
Effects of HO‐3867 on the cell viability of SCC‐9 and HSC‐3 cells. (A) The structure of curcumin analog HO‐3867. (B, C) The viability of SCC‐9 and HSC‐3 cells treated with HO‐3867 (0, 2.5, 5, 10 and 20 µM) for 24 h was detected by MTT assay and CCK‐8 assay, and the effects are illustrated after quantitative analysis. *p < 0.05, compared with the vehicle group

Curcumin and its analogs have been shown their anticancer effects, including suppression of oral squamous cell carcinoma (Table 1). The main goal of this study is to examine whether the novel curcumin analog HO‐3867 exhibits antitumour activity (Figure 1A). We first performed cytotoxicity assay in human oral cancer SCC‐9 and HSC‐3 cells using MTT assay. We found HO‐3867 effectively suppressed SCC‐9 and HSC‐3 cells growth at the dose region from 10 to 20 µM (Figure 1B). Moreover, cell proliferation was assessed by using the CCK‐8 method in SCC‐9 and HSC‐3 cells. As shown in Figure 1C, treatment of cells with HO‐3867 for 24 h significantly decreased the proportion of viable cells in a concentration‐dependent manner. Our results show that HO‐3867 inhibits the cell growth and cell proliferation in human oral cancer SCC‐9 and HSC‐3 cells in vitro.

**TABLE 1:** Molecular actions of curcumin analog on human OSCC cells

Curcumin analog	Cell line	Mechanism of action	Testing dose	References
Curcumin	YD10B	↑reactive oxygen species (ROS) production and autophagy ↑LC3‐II formation and PARP cleavage	1–40 μM	[70]
FLLL‐32	SCC‐9 HSC‐3	↓cell viability ↑apoptosis via caspase‐3/‐8/‐9 and p38 MAPK signalling pathway ↑HO‐1	1–16 μM	[50]
PAC (3,5‐Bis (4‐hydroxy‐3‐methoxybenzylidene)‐N‐methyl‐4‐piperidone)	CA9‐22 gingival epithelial cells (GEC)	↓cell proliferation and colony formation ↑cytotoxicity, intracellular ROS, intracellular glutathione (GSH) activity ↑autophagy by targeting LC3B and p62 ↓epithelial‐to‐mesenchymal transition and inhibits cell migration ↓mitochondrial membrane potential ↑apoptosis via ERK1/2, p38/JNK, NF‐κB and Wnt cellular signalling pathways	1–10 μM	[72]
EF‐24 (diphenyl difluoroketone)	CAL‑27	↓cell viability ↑apoptosis via caspase‐3and 9 ↓phosphorylated forms of MEK1 and ERK	0.1–30 μM	[73]
EF‐24 (diphenyl difluoroketone)	KB	↓cell viability ↑nuclear condensation and fragmentation ↑apoptosis via caspase‐3/‐7/‐9	0.1–100 μM	[74]
DMC (Demethoxycurcumin)	SCC‐9 HSC‐3	↓cell viability ↑G2/M phase arrest ↑apoptosis via caspase‐3/‐8/‐9 and PARP ↓cIAP1/XIAP and activating the p38 MAPK‐HO‐1 axis	12.5–50 μM	[49]
DBA (Dibenzylideneacetone)	Human mucoepidermoid carcinoma (MC3 and YD15)	↓cell viability ↑apoptosis by inhibition of specificity protein 1 (Sp1) protein stability ↑Bim and truncated Bid (t‐Bid) via Sp1 Anti‐tumorigenic activity of DBA (20 mg/kg/day) in an athymic nude mouse xenograft model	5–15 μM	[75]
DBA (Dibenzylideneacetone)	HSC‐4 HSC‐2 YD‐10B SCC‐15	↓cell viability ↑apoptosis through Sp1 degradation ↑increased Bax expression	2.5–10 μM	[71]
trienone 11 (1,7‐bis(3‐hydroxyphenyl)‐1,4,6‐heptatrien‐3‐one)	CLS‐354	↑apoptotic cell death via ROS and caspase‐3/7, ‐8, and ‐9 activations Activates ROS to mediate caspase activation and eventually apoptosis via the intrinsic pathway	0.01–80 μM	[76]
H‐4073	UM‐SCC‐74A UM‐SCC‐1 UM‐SCC‐74B UM‐SCC‐38 UM‐SCC‐47 CAL27	↓cell proliferation, migration, survival and angiogenesis cell proliferation via JAK/STAT3, FAK, Akt and VEGF signalling pathways ↓tumour growth and angiogenesis in SCID mouse xenograft model	2.5–20 μM	[77]

### 3.2 HO‐3867 induces apoptosis and sub‐G1 fraction arrest of SCC‐9 and HSC‐3 cells

**FIGURE 2:**
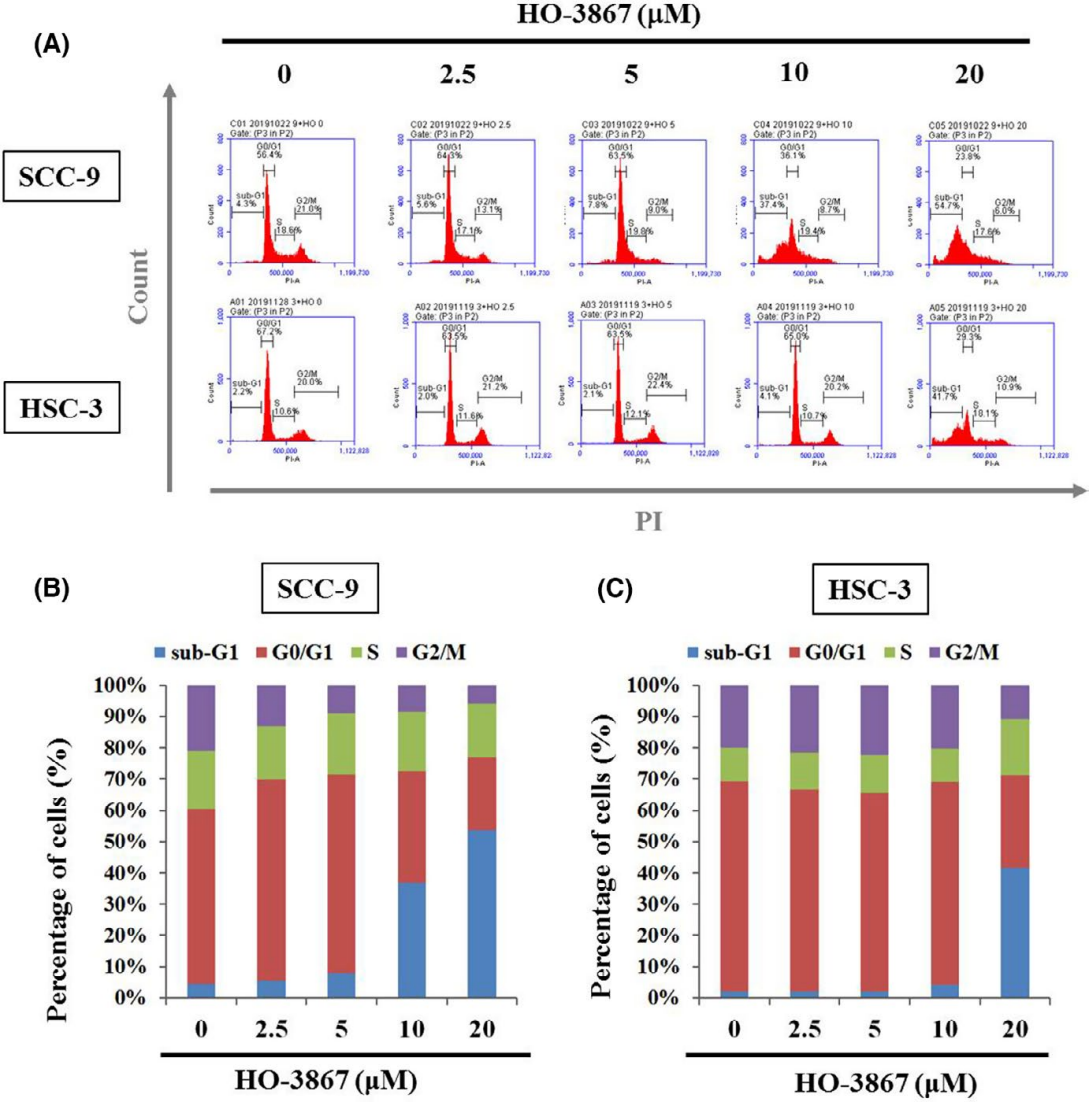
Effects of HO‐3867 on the cell cycle of SCC‐9 and HSC‐3 cells. SCC‐9 and HSC‐3 cells were treated with HO‐3867 (0, 2.5, 5, 10 and 20 µM) for 24 h and then subjected to flow cytometry after (A) PI staining to analyse DNA contents. (B, C) The cell cycle profile of (B) SCC‐9 cells and (C) HSC‐3 cells in flow cytometry was quantified

Given that HO‐3867 potently suppressed cell viability in SCC‐9 and HSC‐3 cells, we assumed that HO‐3867 may affect with cell cycle progression. To examine this hypothesis, we tested the cell cycle progression of oral cancer SCC‐9 and HSC‐3 cells by FACS. Compared to the vehicle control, HO‐3867‐treated SCC‐9 and HSC‐3 cells exhibited a sub‐G1 phase accumulation at the dose of 20 µM (57.7% in SCC‐9 cells and 41.7% in HSC‐3 cells) (Figure 2A–C).

### 3.3 HO‐3867 increases cleaved caspase 3 and decreases cIAP‐1 and XIAP in SCC‐9 and HSC‐3 cells

**FIGURE 3:**
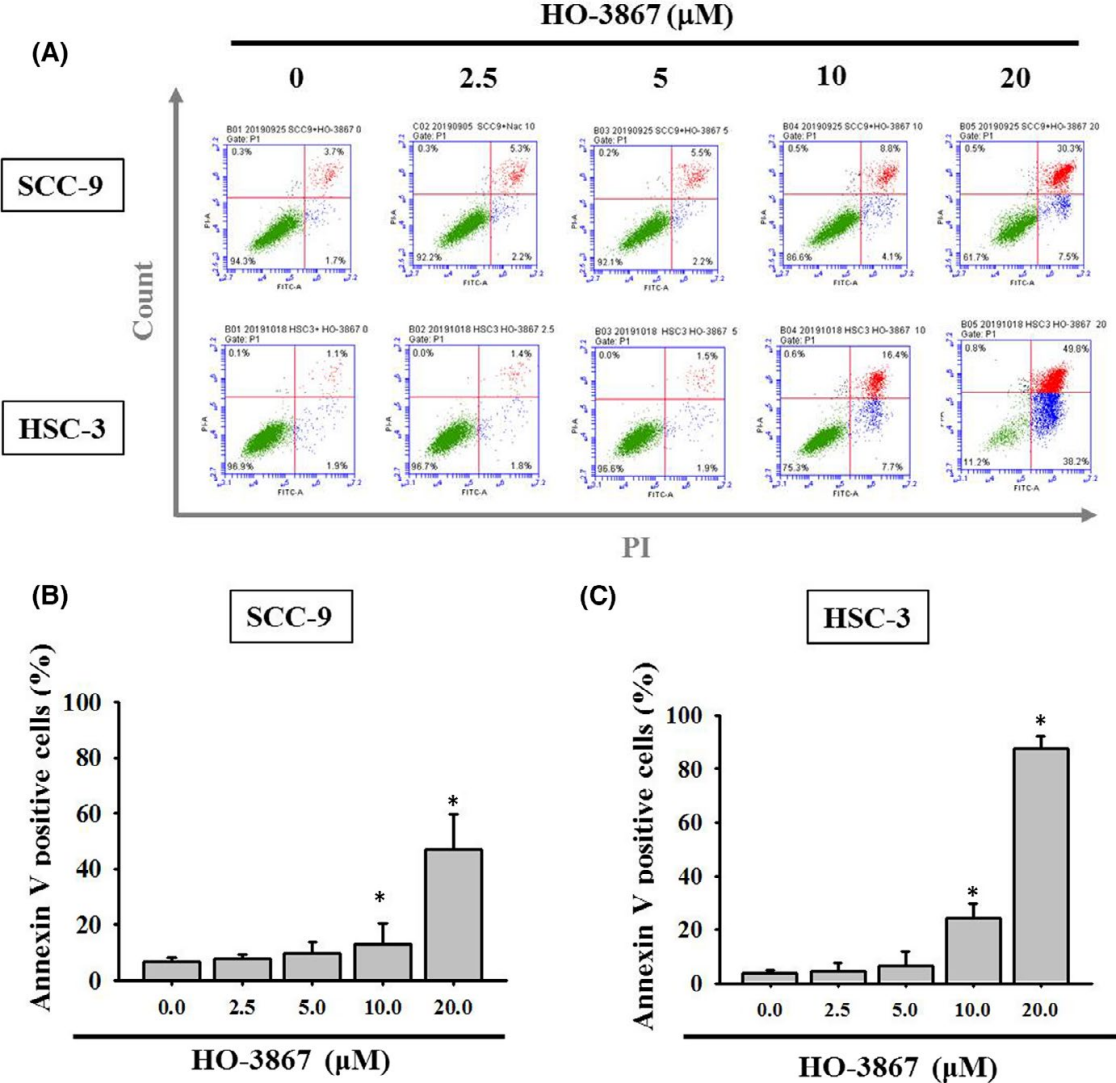
Effects of HO‐3867 on the cell apoptosis in SCC‐9 and HSC‐3 cells. Oral cancer SCC‐9 and oral cancer HSC‐3 cells were treated with HO‐3867 (0, 2.5, 5, 10 and 20 µM) for 24 h and then subjected to (A) flow cytometry to analyse DNA contents. (B, C) Subsequently quantitative analyses of apoptosis of (B) SCC‐9 cells and (C) HSC‐3 cells were summed up. *p < 0.05, compared with the vehicle group

Increased sub‐G1 phase cells in HO‐3867 cells suggest apoptotic pathway may be induced by the treatment of HO‐3867. To test this possibility, we measured and apoptotic cell populations in HO‐3867‐treated SCC‐9 and HSC‐3 cells. Examining the Annexin V positive cells by flow cytometry assay, we found that there were significant inductions of Annexin V positive cells in both lines treated with HO‐3867 (Figure 3A). Remarkedly, at the dose of 20 µM, HO‐3867 induced extremely high amount of cell apoptosis by over 40% in SCC‐9 cells and over 80% in HSC‐3 cells (Figure 3B,C).

### 3.4 Analysis of activating extrinsic and intrinsic apoptotic processes by HO‐3867 in SCC‐9 and HSC‐3 cells

**FIGURE 4:**
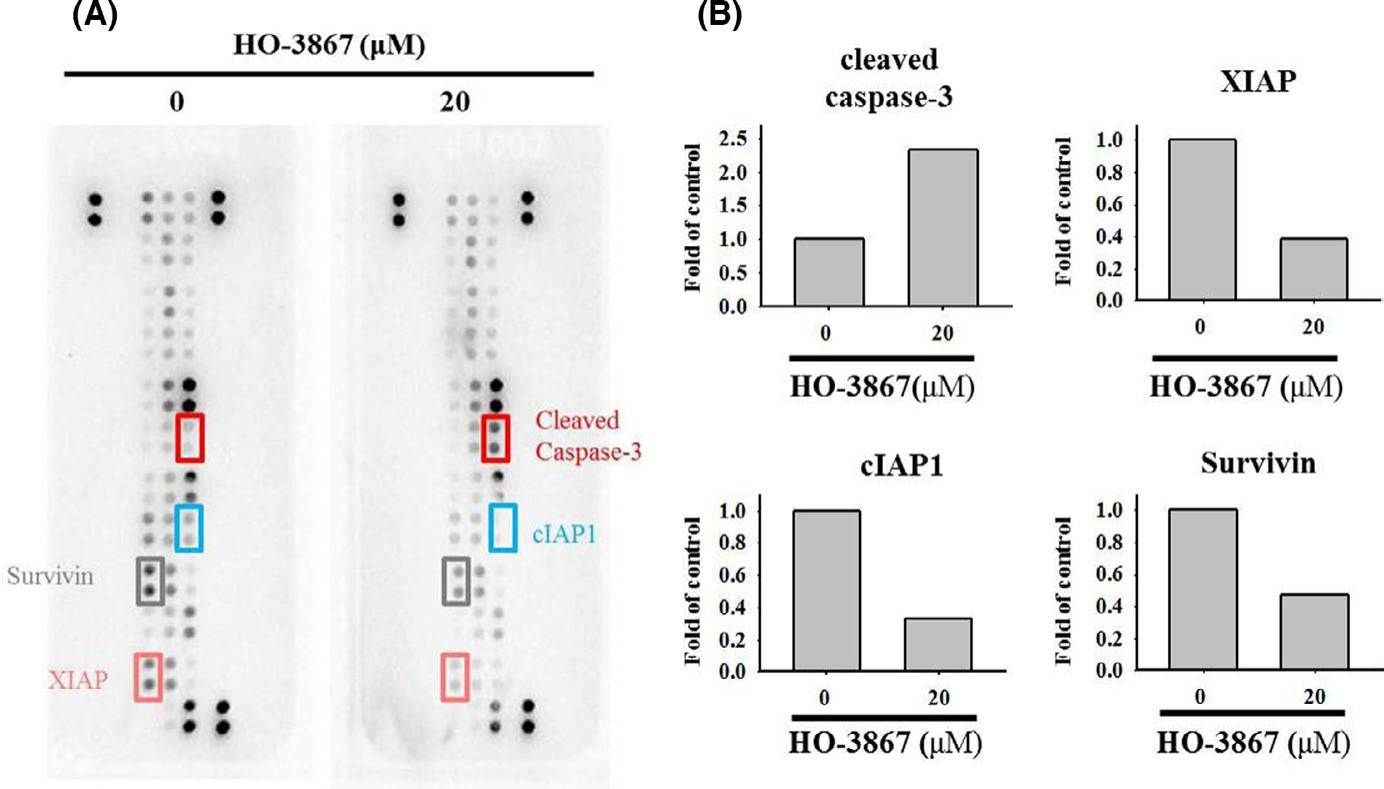
Effects of HO‐3867 on the human apoptosis array and IAPs expression in SCC‐9 cells. (A) After treatment of 20 µM HO‐3867 for 24 h in SCC‐9 cells, the human apoptosis array (ARY009, R&D systems) were employed and the increased cleaved caspase‐3 protein and decreased XIAP, cIAP1 and survivin proteins were exposed to quantitative analysis. (B) Intensity qualifications of cleaved caspase‐3, XIAP, cIAP1 and survivin in HO‐3867‐treated SCC‐9 cells

The next question is how apoptosis was activated in HO‐3867‐treated cells. Since curcumin can increase apoptotic levels through multiple signalling, such as TNF and caspase 8,^44^ we hypothesized HO‐3867 may affect signalling associated with apoptosis or cell survival. To test this hypothesis, we first examined human apoptosis array (ARY009, R&D systems) in SCC‐9 cells. The human apoptosis array contains 35 proteins that associated with apoptotic process as our previous reports.^45^ We identified a serial change in the protein amounts (Figure 4A) We found that cleaved caspase‐3 was increased by approximately 2.3‐fold in HO‐3867‐treated SCC‐9 cells compared to the vehicle control (Figure 4B). Nevertheless, we also found that XIAP, cIAP and Survivin were reduced by 60% upon HO‐3867 (Figure 4B). These results indicate that HO‐3867 induces apoptotic pathway through activating cleaved caspase‐3 in human oral cancer cells.

**FIGURE 5:**
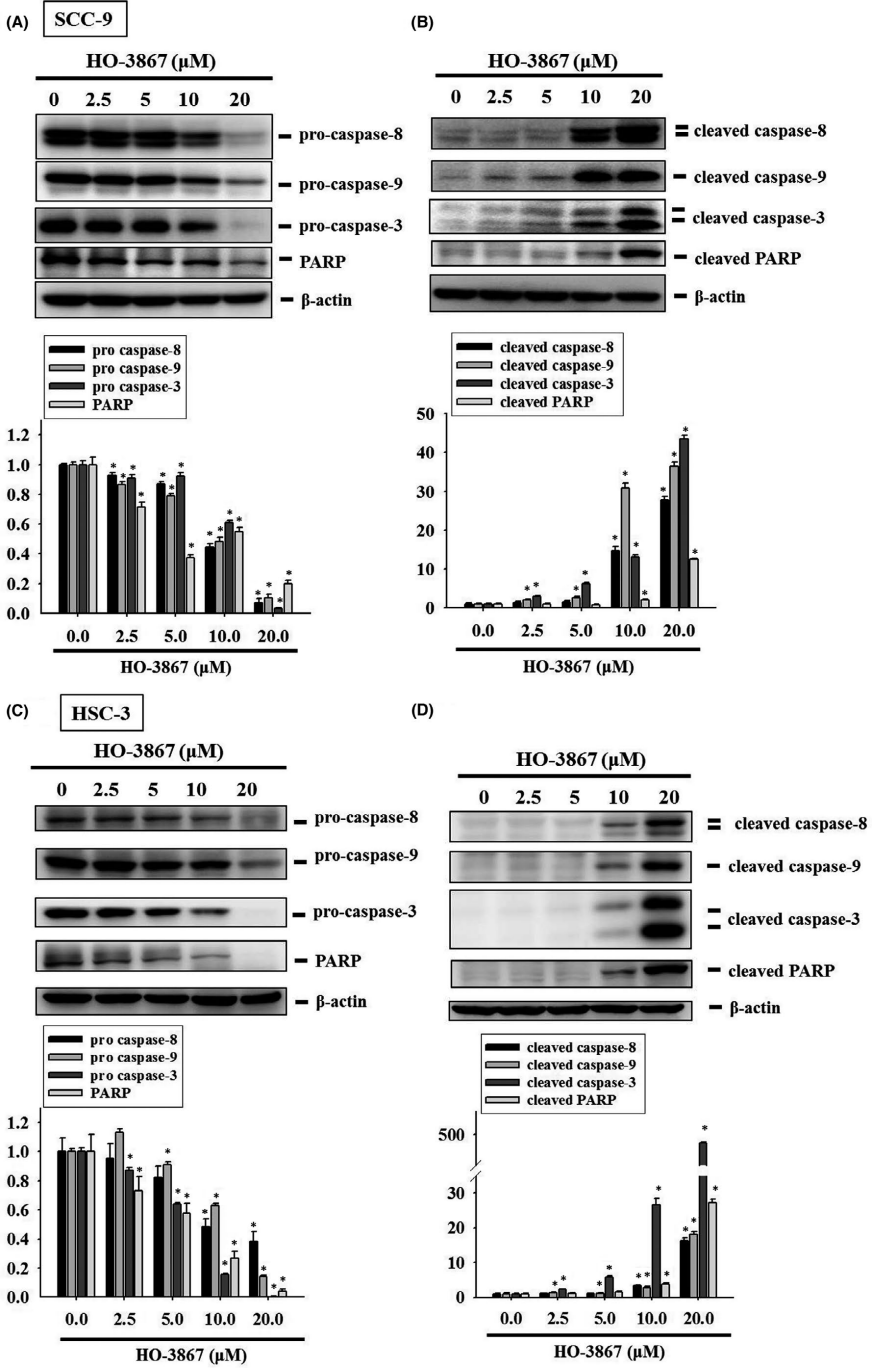
Effects of HO‐3867 on the activation of caspases ‐3, ‐8, and ‐9 in SCC‐9 and HSC‐3 cells. Western blot analysis for (A) caspase‐3, caspase‐8 caspase‐9 and PARP as well as (B) their active forms after various concentrations (0, 2.5, 5, 10 and 20 µM) of HO‐3867 treatment for 24 h in SCC‐9 and (C, D) HSC‐3 cells were measured. All of them were subjected to quantitative analysis. *p < 0.05, compared with the control group

The clarify the capability of HO‐3867 triggering apoptotic pathway through activating cleaved caspase proteins, we measured both total and cleaved forms of apoptotic proteins, including caspase 8, caspase 9, caspase 3 and PARP in HO‐3867‐treated SCC‐9 and HSC‐3 cells. Significantly, treatment of HO‐3867 decreased the pro form of caspase 8, caspase 9, caspase 3 and PARP in SCC‐9 cells (Figure 5A). Treatment of HO‐3867 increased the active form of caspase 8, caspase 9, caspase 3 and PARP in SCC‐9 cells (Figure 5B). Consistently, in HSC‐3 cells, HO‐3867 reduced the pro form of caspase 8, caspase 9, caspase 3 and PARP, and enhanced the active form of caspase 8, caspase 9, caspase 3 and PARP (Figure 5C,D) These results imply that HO‐3867 induces apoptotic pathway through caspase pathway in human oral cancer cells.

### 3.5 HO‐3867 activates extrinsic and intrinsic apoptotic processes via JNK1/2 pathways in SCC‐9 and HSC‐3 cells

**FIGURE 6:**
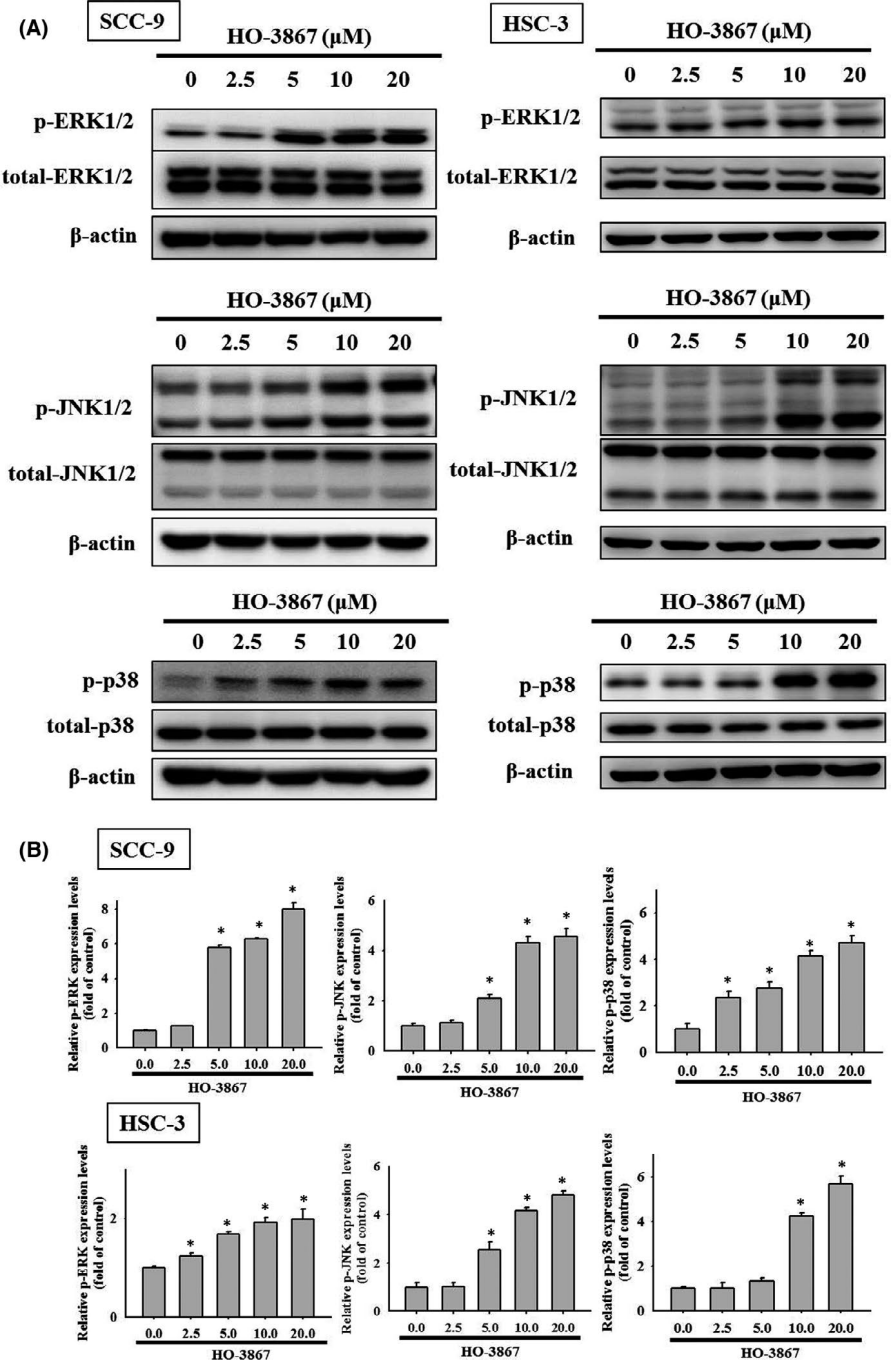
Effects of HO‐3867 on the phosphorylation of MAPK pathway in SCC‐9 and HSC‐3 cells. (A) Expressions of ERK1/2, JNK1/2 and p38, as well as their phosphorylation after various concentrations (0, 2.5, 5, 10 and 20 µM) of HO‐3867 treatment for 24 h in SCC‐9 and HSC‐3 cells, were measured via Western blot analysis. (B) They were subjected to quantitative analysis. *p < 0.05, compared with the vehicle group

**FIGURE 7:**
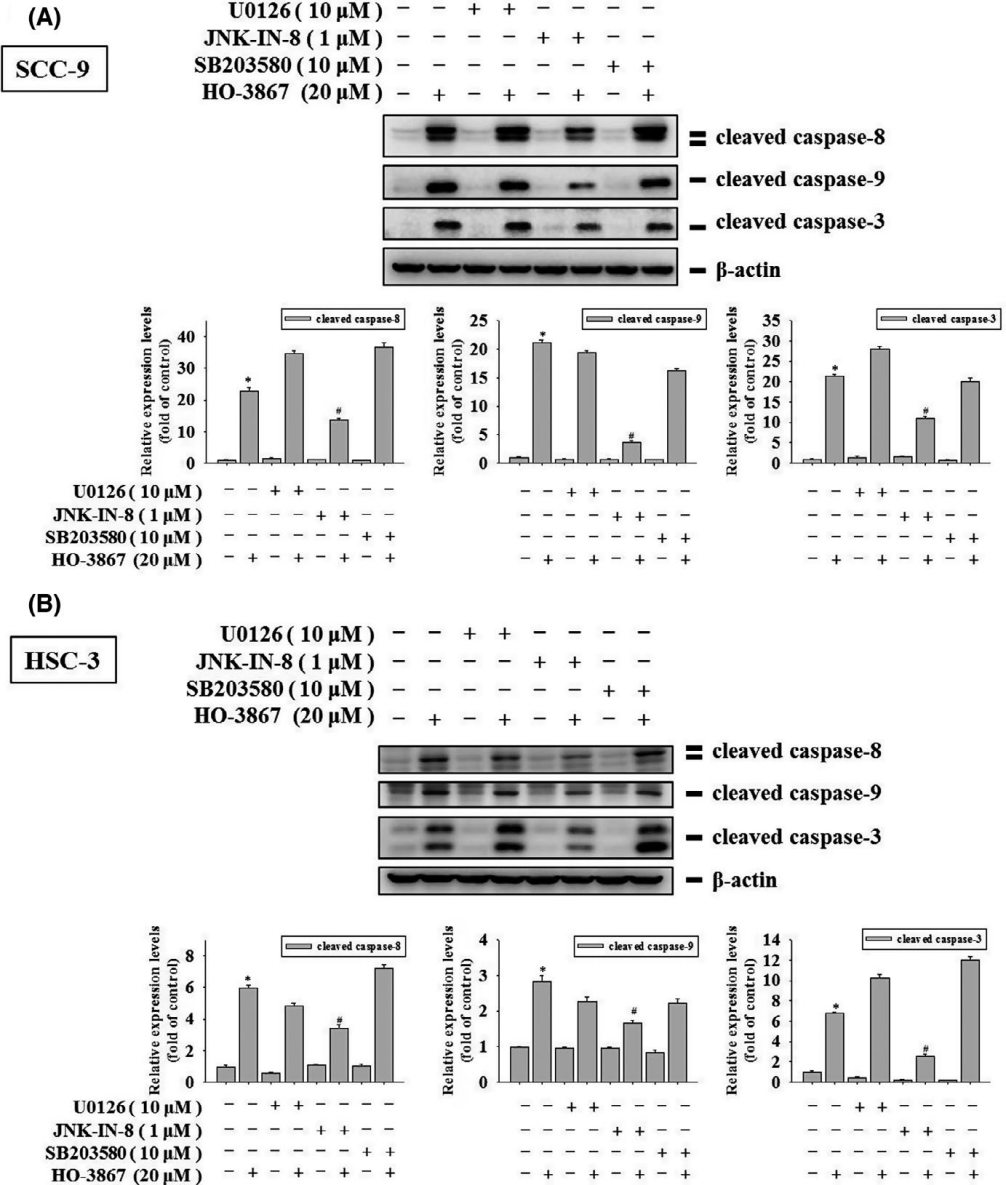
Effects of HO‐3867 and inhibitors of ERK1/2, JNK1/2 and p38 on cleaved caspase‐3, caspase‐8 and caspase‐9 expressions of SCC‐9 and HSC‐3 cells. Expressions of cleaved caspases 3, 8 and 9 after pretreatment with or without 10 µM of U0126 (ERK1/2 inhibitor), 1 µM of JNK‐IN‐8 (JNK1/2 inhibitor) and 10 µM of SB203580 (p38 inhibitor) for 1 h followed by 20 µM or without HO‐3867 treatment for an additional 24 h in (A) SCC‐9 and (B) HSC‐3 cells were measured through Western blot analysis. Next, they were subjected to quantitative analysis. *p < 0.05, compared with the vehicle group. #*p* < 0.05, compared with the HO‐3867‐treated group

Mitogen‐activated protein kinase pathway is known to mediate the apoptotic pathway.^20^ To determine whether the treatment of HO‐3867 could activate MAPK signalling in human oral cancer cells, we detected the phosphorylated levels of ERK1/2, JNK1/2 and p38 in human oral cancer cells SCC‐9 and HSC‐3 cells using immune blot. Upon the treatment of HO‐3867 at the dosages of 2.5–20 µM, phosphorylated ERK1/2 (p‐ERK1/2), p38 (p‐p38) and phosphorylated JNK1/2 (p‐JNK1/2) were enriched (Figure 6A,B), indicating HO‐3867 activates MAPKs pathway in human oral cancer cells. To further digest which MAPK signalling is response to HO‐3867 induced apoptosis, we next measure apoptotic signalling in HO‐3867‐treated human oral cancer cells SCC‐9 and HSC‐3 cells under inhibitions of ERK1/2, JNK1/2 or p38. The inhibitors were U0126,^46^ JNK‐IN‐8^47^ and SB203580^48^ for blocking the phosphorylation of ERK1/2, JNK1/2 and p38 respectively. According to previous studies,^49^, ^50^ after 2‐hour pretreatments of U0126 (10 µM), JNK‐IN‐8 (10 µM) and SB203580 (10 µM), cells were administered with HO‐3867 (20 µM) for another 24 h. We found the treatment of JNK‐IN‐8 was able to attenuate the formation of the active form of caspase 3, caspase 8, caspase 9 and PARP in SCC‐9 cells (Figure 7A). Moreover, as shown in Figure 7B, inhibition of JNK1/2 pathway using JNK‐IN‐8 (10 µM) effectively reduced the apoptotic pathway as the active form of caspase 3, caspase 8, caspase 9 and PARP were reduced in HSC‐3 cells (Figure 7B). Altogether, using inhibitors of JNK1/2 (JNK‐IN‐8), HO‐3867 increases in cleaved caspases 3, 8 and 9 are rescued, but they could not be affected by co‐treatment with the U0126 (ERK1/2 inhibitor) and p38 inhibitor (SB203580). HO‐3867 induces apoptotic pathways in OSCC SCC‐9 and HSC‐3 cells through activating JNK1/2 signalling.

## 4 DISCUSSION

As HO‐3867 is a versatile antitumour agent with targeting STAT3, PTEN and p53 in various cancer types,^22^, ^23^, ^24^, ^25^, ^26^, ^27^, ^28^, ^29^, ^30^, ^31^ we attempted to examine whether HO‐3867 suppress OSCC and to analyse how HO‐3867 triggered cell death. In the present study, we detected that HO‐3867 exhibited great therapeutic effects on OSCC cells, including inducing G2/M cell cycle arrest and apoptotic cell death. Upregulation of the p‐JNK1/2 and downregulation of cIAP1/XIAP/Survivin were critical for HO‐3867‐induced apoptotic cell death in OSCC cells.

It is exciting to find that JNK1/2 signalling is elevated in HO‐3867‐treated OSCC cells. While it is reported that the JNK1/2 signalling and IAPs mediate cell apoptosis,^51^ our study has revealed a novel avenue of modulating JNK1/2 signalling and IAPs using a single compound, HO‐3867. Although inhibitors directly targeting either JNK1/2 or IAPs are not clinical used, development of curcumin and its analogs into clinical is actively processing,^52^ as curcumin functions as an anticancer agent in in vitro, in vivo studies and clinical trials.^53^ However, the detail mechanism of how HO‐3867 activates JNK1/2 and attenuates IAPs in OSCC still unknown.

Over decades, drugs targeting p53, STAT3, ERK1/2, JNK1/2 and p38 are investigated for anticancer propose,^54^, ^55^, ^56^, ^57^, ^58^, ^59^ such as COTI2 for reactivation of mutant p53 to a form with WT properties,^60^ LLL12B blocking STAT3,^61^ LY3214996 targeting ERK1/2,^62^ AS602801 suppressing JNK^63^ and BIRB796 targeting p38.^64^ Therefore, as HO‐3867 is reported to target p53,^34^ STAT3,^29^ JNK1/2 and IAPs, HO‐3867 would be a potential therapeutic approach for treatment of OSCC or other types of cancers that may have dysregulation of p53, STAT3, JNK1/2 and IAPs.

As a versatile compound, HO‐3867 has been examined its potential to be a treatment for many diseases, such as breast cancer,^34^ ovarian cancer,^22^, ^25^, ^26^, ^30^, ^35^, ^65^ pancreatic cancer^29^, ^31^ and endometrial cancer.^28^ HO‐3867 is found not only induce apoptosis in ovarian cancer cells^25^, ^65^ but also to repress the migration and invasion of ovarian cancer cells by inhibiting the expression or activity of FAS, FAK, VEGF and their downstream protein levels.^25^ Moreover, HO‐3867 has been evaluated for the treatment of pulmonary hypertension,^66^ pulmonary hypertension secondary to left‐heart failure^33^ and arterial restenosis.^32^ Together, these researches indicate that HO‐3867 has highly potential to be developed into an anticancer agent or a regimen for other diseases.

Curcumin and its analogs have been shown their anticancer effects in vitro and in vivo,^67^ including suppression of oral squamous cell carcinoma.^68^ Through targeting EGFR mediated AKT, ERK1/2 and STAT3 pathways, curcumin inhibits SCC‐25 cell growth at the dosage range of 10–80 µM.^69^ Moreover, curcumin promoted apoptosis by inducing cleaved caspase 3 and cleaved PARP in YD10B OSCC cells at the dose of 10 µM.^70^ Interestingly, several analogs of curcumin are identified their antitumour activity through induction of apoptosis in OSCC.^71^, ^72^ In HSC‐4 and HSC‐2 human oral cancer cells lines, Dibenzylideneacetone inhibits cell viability by triggering apoptosis at the dose of 5–10 µM.^71^ PAC (3,5‐Bis (4‐hydroxy‐3‐methoxybenzylidene)‐N‐methyl‐4‐piperidone) is recently reported to reduce cell survival through promote apoptosis and autophagy by activating NF‐κB, MAPK, Wnt, caspase‐3/9 and PARP1 at the dose of 5 µM in oral cancer CA9‐22 cells.^72^ In this study, we show that the curcumin analog HO‐3867 exhibits anti‐OSCC activity by inducing apoptosis at the similar dose range (2.5–20 µM), suggesting that HO‐3867 is comparably potent to OSCC as curcumin and other analogs.

In conclusion, we have revealed that the curcumin analog HO‐3867 suppresses OSCC growth via inducing cell cycle arrest and apoptosis. As inhibition of apoptosis is a hallmark of cancer progression, we found HO‐3867 triggers OSCC cell apoptosis via promoting cleaved caspase‐3 through JNK1/2 signalling. As the results, HO‐3867 has high potential to improve the outcome of treatment of OSCC.

## CONFLICT OF INTEREST

The authors declare that there is no conflict of interest.

## AUTHOR CONTRIBUTIONS

**Chi‐Wei Chen:** Conceptualization (equal); Writing – original draft (equal); Writing – review & editing (equal). **Ming‐Ju Hsieh:** Methodology (equal); Resources (equal). **Po‐Chung Ju:** Methodology (equal). **Yi‐Hsien Hsieh:** Methodology (equal). **Chun‐Wen Su:** Methodology (equal). **Yen‐Lin Chen:** Investigation (equal); Writing – review & editing (equal). **Shun‐Fa Yang:** Conceptualization (equal); Writing – original draft (equal); Writing – review & editing (equal). **Chiao‐Wen Lin:** Conceptualization (equal); Writing – original draft (equal); Writing – review & editing (equal).

## Data Availability

The data used to support the findings of the present study are available from the corresponding author upon request.
